# Supplementary material for: Relationships between Iraqi Rice Varieties at the Nuclear and Plastid Genome Levels
Source: Plants (Basel). 2019 Nov 7;8(11):481. doi: 10.3390/plants8110481 (PMC6918272; doi:10.3390/plants8110481)
Supplement: Supplementary file 1 [file plants-08-00481-s001.pdf]

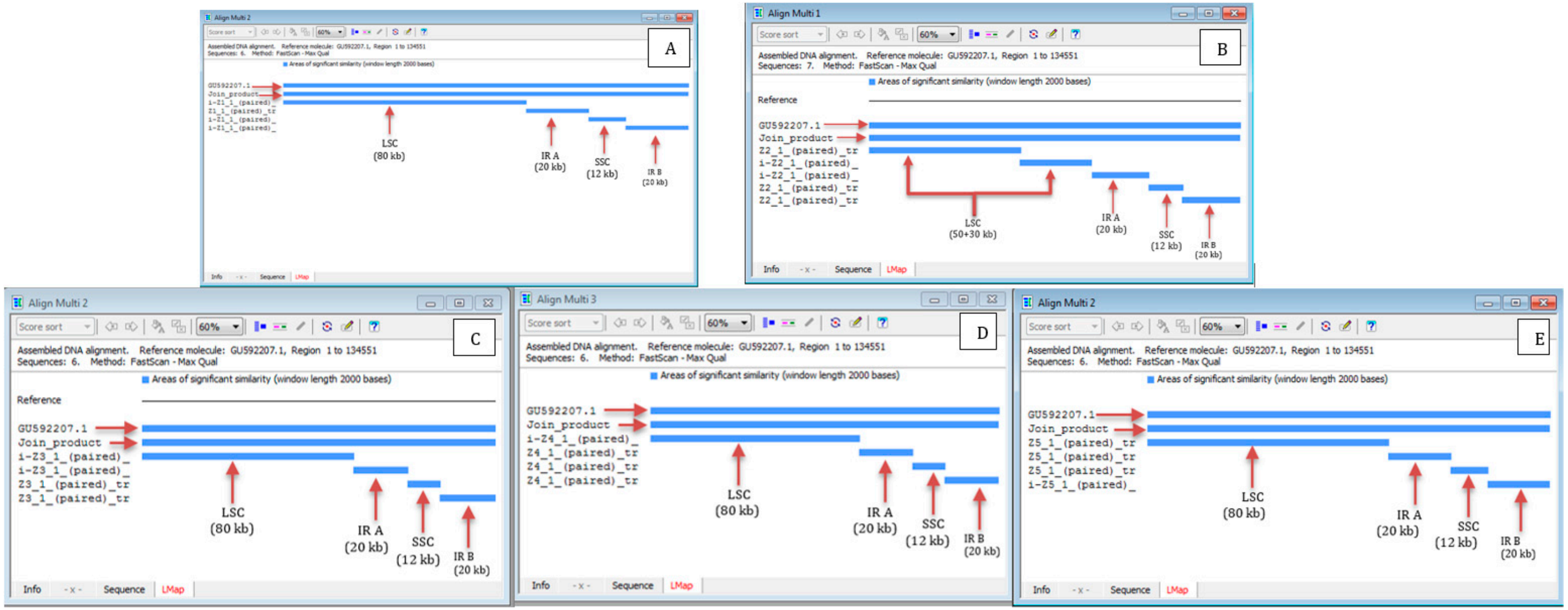

Figure S1: The results of *de novo* Assembly on Clone Manager Professional 9.0 software.

The results represented by the Chloroplast contigs with their length (kb) of five Iraqi varieties, **A**: Amber33, **B**: Furat, **C**: Yasmin, **D**: Buhooth1 and **E**: Amber al-Baraka; **LSC**: Large Single Copy, **IR A**: Inverted Repeat A, **SSC**: Small Single Copy, **IR B**: Inverted Repeat B; **Join product**: Contigs alignment product; **GU592207.1**: Reference (*O. sativa* subsp. Japonica Nipponbare GU592207.1).

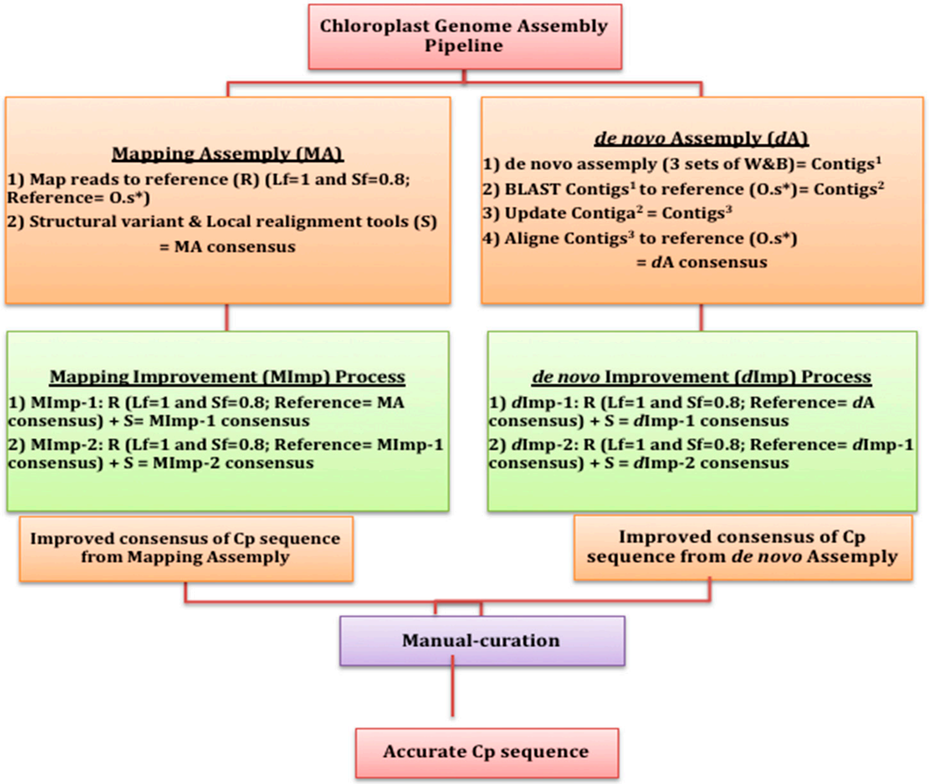

Figure S2: Illustration of the Chloroplast Genome Assembly Pipeline.

* *O. sativa* sub sp. japonica Nipponbare 'GenBank: GU592207.1'

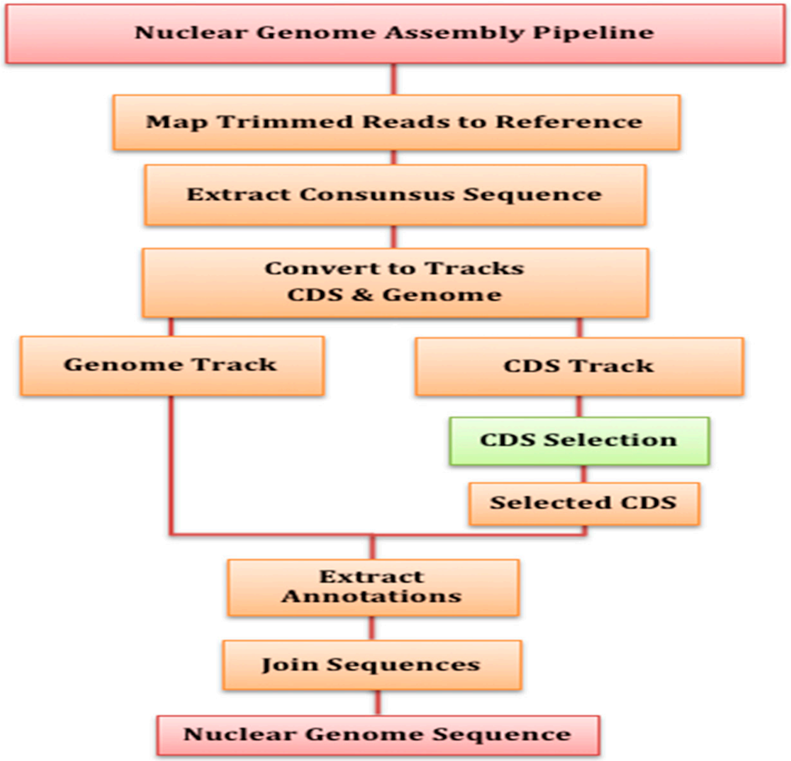

Figure S3: Illustration of the Nuclear Genome Assembly Pipeline.

Table S1: Summary of the output of sequencing and downloading (Raw Data) and trimming processes.

Varieties	Raw Data					After Trimming (0.01)			
	Total number of nucleotides	No. of reads	Reads (%)	Avg. length (bp)	Cov (×)	No. of reads	Reads (%)	Avg. length (bp)	Cov (×)
**Amber33**	9 386 089 332	62 159 532	100	151	25	56 823 748	91	128	20
**Furat**	14 014 203 696	92 809 296	100	151	38	85 740 259	92	130	30
**Yasmin**	9 711 320 078	64 313 378	100	151	26	59 256 230	92	129	21
**Buhooth1**	8 710 458 556	57 685 156	100	151	23	52 895 284	92	129	18
**Amber al-Baraka**	9 036 149 550	59 842 050	100	151	24	54 333 116	91	127	19
**B243**	6 619 301 958	79 750 626	100	83	18	77 666 434	93	79	17
**CX165**	6 679 723 966	80 478 602	100	83	18	77 948 490	92	79	17
**CX352**	7 095 277 078	85 485 266	100	83	19	83 140 722	92	79	18
**CX10**	9 739 096 330	117 338 510	100	83	26	112 875 336	91	79	24
**CX368**	5 138 139 402	61 905 294	100	83	14	60 147 030	92	79	13
**IRIS_313-10670**	4 271 549 516	51 464 452	100	83	11	50 105 006	93	80	11
**IRIS_313-11153**	4 224 233 706	50 894 382	100	83	11	49 416 724	92	79	10
**IRIS_313-11479**	4 861 119 598	58 567 706	100	83	13	57 163 653	94	80	12
**IRIS_313-11152**	3 598 864 894	43 359 818	100	83	10	42 050 703	92	79	9
**CX129**	5 846 882 046	70 444 362	100	83	16	68 259 826	92	79	14
**CX25**	5 758 640 098	69 381 206	100	83	15	67 540 020	93	79	14
**CX104**	6 483 646 924	78 116 228	100	83	17	74 634 286	90	78	16
**CX227**	7 867 758 078	94 792 266	100	83	21	92 284 477	94	80	20
**CX140**	7 419 659 670	89 393 490	100	83	20	85 982 557	91	78	18
**IRIS_313-10073**	4 794 844 596	57 769 212	100	83	13	57 027 465	96	81	12
**IRIS_313-10549**	4 833 856 256	58 239 232	100	83	13	55 568 578	89	78	12
**IRIS_313-11021**	5 344 796 454	64 395 138	100	83	14	62 605 912	93	79	13
**IRIS_313-11026**	4 093 770 156	49 322 532	100	83	11	47 912 979	92	79	10
**IRIS_313-8656**	4 454 294 766	53 666 202	100	83	12	52 664 918	95	81	11
**IRIS_313-11829**	7 166 823 078	86 347 266	100	83	19	83 515 941	91	78	17
**IRIS_313-10380**	5 475 067 210	65 969 272	100	83	15	63 883 416	92	78	13
**CX59**	6 132 312 406	73 883 282	100	83	16	72 498 885	95	81	16
**IRIS_313-10373**	3 992 905 568	48 107 296	100	83	11	46 679 053	92	79	10
**CX243**	7 168 608 408	86 368 776	100	83	19	83 656 775	93	79	18
**IRIS_313-9505**	4 692 559 712	56 536 864	100	83	13	55 104 301	92	78	12
**CX126**	5 127 291 302	61 774 594	100	83	14	59 939 466	93	79	13
**IRIS_313-10718**	8 126 233 358	97 906 426	100	83	22	95 153 374	91	78	20
**IRIS_313-9949**	5 559 042 196	66 976 412	100	83	15	65 074 992	91	78	14
**IRIS_313-11248**	3 688 302 374	44 437 378	100	83	10	42 826 311	90	77	9
**CX106**	8 820 564 380	106 271 860	100	83	24	101 821 850	90	78	21
**B009**	6 579 238 356	79 267 932	100	83	18	76 645 745	92	79	16
**CX37**	4 118 516 772	49 620 684	100	83	11	48 485 263	93	79	10

**O.glaberrima-PRJNA13765**	10 653 849 150	142 051 322	100	75	28	134 949 944	89	70	25

Each variety represented by the total number of nucleotides, total number of reads (No. of reads), average read length (Avg. Length bp) and average read coverage across the genome (Cov×), for five Iraqi verities and 32 domesticated Asian accessions and one domesticated African rice as an out-group downloaded from SAR-NCBI.

Table S2: Summary of Mapping Assembly process using three different setting of Length fraction and Similarity Fraction.

Varieties ID	Variations	LF 0.8, SF 0.8	LF 1, SF 0.8	LF 1, SF 0.9
**Amber33**	**Length (bp)**	134552	134550	134550
	**Mismatch**	20	20	20
	**Gap**	7	6	6
	**No. Mapped Reads**	3 019 921	3 002 212	2 986 518
**Furat**	**Length (bp)**	134555	134553	134554
	**Mismatch**	73	73	75
	**Gap**	24	23	22
	**No. Mapped Reads**	8 031 381	7 992 122	7 950 234
**Yasmin**	**Length (bp)**	134553	134553	134554
	**Mismatch**	72	72	74
	**Gap**	23	23	22
	**No. Mapped Reads**	5 642 013	5 618 617	5 593 804
**Buhooth1**	**Length (bp)**	134553	134553	134552
	**Mismatch**	4	4	4
	**Gap**	2	2	1
	**No. Mapped Reads**	4 928 685	4 910 697	4 894 042
**Amber al-Baraka**	**Length (bp)**	134554	134554	134555
	**Mismatch**	73	73	75
	**Gap**	22	22	21
	**No. Mapped Reads**	3 817 251	3 796 146	3 774 843

The consensus length (Length (bp)), number of mapped reads (No. Mapped Reads), mismatches and gaps in the sequences of mapping consensus of the chloroplast genome for Iraqi varieties derived from Mapping Assembly (MA) process against *O. sativa* sub sp. japonica Nipponbare ‘GenBank: GU592207.1’ under three various

mapping settings of Length fraction (LF) and Similarity Fraction (SF).Table S3: Comparison between Mapping and *de novo* assembly in the number of variations in the chloroplast-genome.

**Variation type**	**Amber 33**		**Furat**		**Yasmin**		**Buhooth1**		**Amber al-Baraka**	
	**MA**	***d*A**	**MA**	***d*A**	**MA**	***d*A**	**MA**	***d*A**	**MA**	***d*A**
**Length (bp)**	134536	134276	134459	134163	134492	134561	134550	134548	134492	134552
**SNPs**	8	8	44	44	43	43	2	2	45	44
**MNPs**	12	12	28	28	28	28	2	2	28	28
**Insertion**	4	4	10	11	14	14	1	2	11	12
**Deletion**	4	7	15	19	13	13	2	1	14	14
**Total**	28	31	97	102	98	98	7	7	98	98

Details of variants among Iraqi varieties chloroplast genomes and *O. sativa* subsp. Japonica Nipponbare GU592207.1 in both Mapping (MA) and *de novo* (*d*A) assembly.

Table S4: Details of the polymorphisms identified in aligned chloroplast-genomes using the "**variant/SNP detection**" tool.

No	Position	Chloroplast regions	Polymorphism Type	Length	Original Nucleotide(s)	Variant Nucleotide(s)	Group
1	412	LSC	SNP	1	T	C	In
2	3098	LSC	SNP	1	C	T	Bas
3	4547	LSC	SNP	1	G	T	In
4	5014	LSC	Ins	7	-	TCCTTTA	In
5	6245	LSC	Del	1	T	-	In
6	6283	LSC	SNP	1	T	C	In & Bas
7	6609	LSC	SNP	1	G	T	In
8	7135	LSC	SNP	1	T	C	In
9	8128	LSC	SNP	1	A	G	In
10	8543	LSC	Del	69	TTTTCAGAAT<br>CCTATTTTTG<br>TTCTTATACC<br>CATGCAATAG<br>AGAGCGAGTG<br>GGAAAAGGGA<br>GGTTACTTT	----------------------<br>----------------------<br>----------------------<br>-------	In
11	12498	LSC	SNP	1	A	G	In
12	12674	LSC	Del	4	AGGG	-	In
13	12801	LSC	SNP	1	G	A	In
14	12821	LSC	MNP	2	TA	CC	In
15	13470	LSC	SNP	1	A	T	In
16	14014	LSC	Del	2	AC	--	In
17	14235	LSC	SNP	1	A	G	In & Bas
18	15018	LSC	SNP	1	C	T	Bas
19	15911	LSC	SNP	1	T	A	In
20	16674	LSC	Ins	1	-	A	In
21	16905	LSC	SNP	1	C	T	Bas (4)
22	16908	LSC	SNP	1	A	G	Bas (4)
23	17206	LSC	SNP	1	A	G	In
24	17384	LSC	Del	6	AAATAG	------	In & Bas
25	17749	LSC	MNP	2	GG	AA	In
26	17814	LSC	Ins	32	--------------------------------	TTAACAAATT<br>CTTAGAGTAT<br>TTCTGGTAGAA<br>T	In
27	18482	LSC	SNP	1	T	G	In
28	18523	LSC	SNP	1	G	A	In
29	20589	LSC	SNP	1	T	A	In
30	28021	LSC	SNP	1	G	T	In
31	29115	LSC	SNP	1	A	G	In
32	35383	LSC	SNP	1	G	A	In
33	43902	LSC	Ins	1	-	A	In
34	43902	LSC	Del	1	A	-	TrpJ (4)
35	46092	LSC	Del	5	TTATA	-	In
36	46183	LSC	Del	1	T	-	In
37	46183	LSC	Ins	1	-	T	TrpJ (4)

38	46541	LSC	Del	6	AAAGAA	-	In
39	47218	LSC	Ins	1	-	T	In
40	47709	LSC	SNP	1	A	C	In & Bas & TrpJ (4)
41	49849	LSC	SNP	1	C	T	In
42	50244	LSC	SNP	1	A	C	In
43	51344	LSC	SNP	1	T	A	In
44	52142	LSC	SNP	1	C	T	In
45	53515	LSC	SNP	1	C	T	In
46	54921	LSC	SNP	1	G	A	In & Bas & TrpJ (4)
47	55673	LSC	MNP	6	GAAAAA	TTTTTC	In & Bas
48	55796	LSC	SNP	1	G	T	In
49	56597	LSC	SNP	1	T	C	In
50	56870	LSC	SNP	1	C	T	In
51	57036	LSC	Del	16	TTTTTTTTAG AATACT	----------------	In & Bas
52	57070	LSC	Del	1	A	-	In & Bas
53	57080	LSC	Ins	5	-	ATACT	In
54	57654	LSC	Ins	5	-	AAAGT	In
55	60871	LSC	Ins	5	-	TTGTA	In
56	61004	LSC	SNP	1	T	G	In
57	62529	LSC	MNP	8	CTTGGTCT	AGACCAAG	In
58	64174	LSC	SNP	1	C	A	In
59	65624	LSC	Ins	1	-	T	Bas
60	65622	LSC	Del	2	TT	--	In
61	66410	LSC	SNP	1	A	G	In
62	69357	LSC	SNP	1	C	T	In
63	74280	LSC	SNP	1	A	C	In & Bas
64	74919	LSC	SNP	1	C	T	TrpJ (4)
65	75990	LSC	Del	1	T	-	*ind*
66	76242	LSC	Del	1	A	-	In
67	76583	LSC	Del	1	T	-	In & Bas & TrpJ (4)
68	76607	LSC	MNP	2	TC	GA	Bas
69	77735	LSC	Ins	3	---	TGG	In
70	77806	LSC	SNP	1	G	A	In
71	79483	LSC	SNP	1	C	T	In & Bas & TrpJ
72	80620	IR A	Ins	2	--	TT	In & Bas
72	80620	IR A	Ins	1	--	T	TrpJ
73	93088	IR A	MNP	3	AAA	TTT	In
74	102942	SSC	SNP	1	C	T	In
75	104302	SSC	SNP	1	G	A	In
76	104540	SSC	Ins	4	----	CAAA	In & Bas & TrpJ (4)
77	104579	SSC	SNP	1	C	A	In
78	105791	SSC	MNP	4	AAGC	GCTT	In &Bas
79	106364	SSC	SNP	1	G	T	In
80	108285	SSC	SNP	1	G	A	In
81	108678	SSC	SNP	1	C	A	In

82	109158	SSC	SNP	1	A	G	In
83	113100	SSC	SNP	1	G	T	In
84	122065	IRB	MNP	3	TTT	AAA	In
85	134545	IR B	Ins	1	--	A	TrpJ
85	134545	IR B	Ins	2	--	AA	In & Bas

The polymorphisms identified in aligned chloroplast-genomes using the "variant/SNP detection" too by Geneious software version 9.1.8; BioMatters, USA. Chloroplast regions: **LSC**: large single copy, **SSC**: small single copy, and **IR A** or **B**: inverted repeat A and B; Polymorphism types: **SNP**: single nucleotide polymorphism, **MNP**: multi nucleotide polymorphism, **Ins**: insertions, and **Del**: deletions (Del); Group: **In**: Indica (including *indica* and *aus* ecotype), **Bas**: Basmati, TrpJ: tropical japonica, **Bas (4)**: Part of Basmati group (4 accessions, including three accessions were from Pakistan and one from Iran), **TrpJ (4)**: Four accessions from tropical japonica (TrpJ) subpopulation, and **ind**: *indica* ecotype.

Table S5: Distance matrix corresponding to the number of non-identical bases in the sequences of domesticated-rice chloroplast-genomes.

	1	2	3	4	5	6	7	8	9	10	11	12	13	14	15	16	17	18	19	20	21	22	23	24	25	26	27	28	29	30	31	32	33	34	35	36	37	38	39
**1**		5	6	6	4	6	8	6	5	6	5	7	5	7	131	131	129	129	132	132	132	131	129	256	126	124	189	111	112	111	111	110	110	113	111	134	111	122	119
**2**			2	2	7	3	5	9	1	10	1	3	1	3	134	128	126	126	129	129	129	135	129	260	122	120	186	109	108	111	111	111	111	114	107	131	111	126	122
**3**				0	6	1	3	8	1	9	1	1	1	4	133	127	125	125	128	128	128	134	128	259	121	119	185	108	107	110	110	112	110	113	108	130	110	125	122
**4**					6	1	3	8	1	9	1	1	1	4	133	127	125	125	128	128	128	134	128	259	121	119	185	108	107	110	110	112	110	113	108	130	110	125	122
**5**						5	7	4	7	4	7	6	7	8	129	131	129	129	132	132	132	130	129	254	126	124	189	111	112	111	111	112	110	113	113	134	111	121	121
**6**							2	7	2	9	2	1	2	4	133	127	125	125	128	128	128	133	128	258	122	120	185	107	108	110	110	111	109	112	109	130	110	125	122
**7**								9	4	11	4	3	4	6	135	129	127	127	130	130	130	135	130	260	124	122	187	109	110	112	112	113	111	114	111	132	112	127	124
**8**									9	8	9	8	9	10	126	127	125	125	128	128	128	126	125	251	122	120	185	113	114	113	113	114	112	115	115	130	113	118	123
**9**										10	0	2	0	3	134	128	126	126	129	129	129	135	129	260	122	120	186	109	108	111	111	111	111	114	107	131	111	126	121
**10**											10	10	10	12	131	135	133	133	136	136	136	134	132	258	129	127	193	115	115	114	114	116	114	113	116	138	114	121	124
**11**												2	0	3	134	128	126	126	129	129	129	135	129	260	122	120	186	109	108	111	111	111	111	114	107	131	111	126	121
**12**													2	4	133	127	125	125	128	128	128	134	129	259	122	120	185	108	108	111	111	112	110	113	109	130	111	125	122
**13**														3	134	128	126	126	129	129	129	135	129	260	122	120	186	109	108	111	111	111	111	114	107	131	111	126	121
**14**															135	129	127	127	130	130	130	136	131	261	124	122	187	110	110	113	113	112	112	115	109	132	113	127	123
**15**																15	13	13	5	5	5	4	8	146	17	15	89	33	33	33	33	34	32	35	34	7	33	13	77
**16**																	3	3	10	10	10	16	11	156	19	17	80	27	27	30	30	31	29	32	28	12	30	22	77
**17**																		0	8	8	8	14	9	154	17	15	78	25	25	28	28	29	27	30	26	10	28	20	74
**18**																			8	8	8	14	9	154	17	15	78	25	25	28	28	29	27	30	26	10	28	20	74
**19**																				0	0	6	6	149	12	10	84	28	28	31	31	32	30	33	29	2	31	18	78
**20**																					0	6	6	149	12	10	84	28	28	31	31	32	30	33	29	2	31	18	78
**21**																						6	6	149	12	10	84	28	28	31	31	32	30	33	29	2	31	18	78
**22**																							8	147	15	16	90	33	34	33	33	33	31	35	35	4	33	17	78
**23**																								148	14	12	86	28	28	27	27	29	27	30	29	8	27	19	77
**24**																									142	140	141	158	159	158	158	159	157	160	160	151	158	141	203
**25**																										2	78	22	21	24	24	25	23	27	22	11	24	12	72
**26**																											76	20	19	22	22	24	22	25	20	12	22	10	70
**27**																												85	85	88	88	89	87	90	86	86	88	81	135
**28**																													1	3	3	4	2	5	2	30	3	24	66
**29**																														3	3	5	3	6	1	30	3	24	65
**30**																															0	2	2	5	4	33	0	24	65
**31**																																2	2	5	4	33	0	24	65
**32**																																	2	6	4	33	2	25	65
**33**																																		4	4	31	2	23	65
**34**																																			7	35	5	24	68
**35**																																				31	4	25	64
**36**																																					33	20	80
**37**																																						24	65
**38**																																							69
**39**																																							

The heatmap demonstrates the individual values in a distance matrix as colours: green cells indicate great distance; yellow, orange and red cells show gradually closer distances **1:**B243, **2:**CX368, **3:**IRIS_313-11152, **4:**IRIS_313-9505, **5:**IRIS_313-10549, **6:**Yasmin, **7:**Furat, **8:**IRIS_313-10380, **9:**CX126, **10:**Amber al-Baraka, **11:**CX37, **12:**CX10, **13:**CX25, **14:**CX227, **15:**CX165, **16:**CX352, **17:**IRIS_313-10073, **18:**CX243, **19:**Ref-GU592207.1, **20:**IRIS_313-11153, **21:**IRIS_313-10373, **22:**IRIS_313-11829, **23:**Buhooth1, **24:**IRIS_313-11479, **25:**IRIS_313-9949, **26:**CX129, **27:**IRIS_313-11248, **28:**IRIS_313-10670, **29:**Amber33, **30:**IRIS_313-8656, **31:**IRIS_313-11026, **32:**IRIS_313-11021, **33:**IRIS_313-10718, **34:**B009**, 35:**CX59, **36:**CX140, **37:**CX104, **38:**CX106, **39:**O.glaberrima-PRJNA13765.
